# Optical Coherence Tomography-Based Diagnosis and Monitoring of Systemic Lupus Erythematosus-Associated Choroidopathy

**DOI:** 10.7759/cureus.29432

**Published:** 2022-09-21

**Authors:** Salil Mehta, Hemant Mehta

**Affiliations:** 1 Ophthalmology, Lilavati Hospital, Mumbai, IND; 2 Nephrology, Lilavati Hospital, Mumbai, IND

**Keywords:** bacillary layer detachment, plasmapheresis, choroidopathy, nephritis, systemic lupus erythematosus

## Abstract

A 55-year-old female presented with bilateral lower limb swelling and facial swelling along with a decreased frequency of micturition. Baseline investigations revealed an elevated serum creatinine and blood urea nitrogen. Subsequent investigations revealed a positive antinuclear antibodies (ANA) +++ (titer 1:10,000, serum immunofluorescence method) and positive anti-double-stranded DNA (dsDNA) testing. A detailed ophthalmic evaluation was performed. Her visual acuity was 6/36 improving to 6/18 in either eye. The near vision was found to be N36 without any further improvement. A dilated fundus examination revealed multiple yellowish lesions throughout the posterior pole consistent with pockets of subretinal fluid in the right eye. A swept-source optical coherence tomography (OCT) was performed that revealed the presence of fluid in both the intraretinal and subretinal spaces. A bacillary layer detachment, with the accumulation of fluid in the intraretinal space, was noted. Similar fundus findings were seen in the left eye. A decision was made to employ plasmapheresis (PLEX) along with her routine thrice-weekly hemodialysis. Additionally, the systemic steroids were continued. At her third follow-up (day 22), her vision had improved to 6/9 unaided bilaterally and to N6 with the appropriate correction. There was a near-complete regression of the exudative retinal detachments bilaterally with pigmentary changes. The OCT scans revealed significant regression of the serous retinal and retinal pigment epithelium (RPE) detachments with a thin rim of residual subretinal fluid. Fundus examination and OCT studies established the diagnosis of systemic lupus erythematosus (SLE) choroidopathy and guided its further management with systemic immunosuppression, hemodialysis and plasmapheresis. There was a rapid resolution of the retinal and choroidal findings with visual recovery over the next month.

## Introduction

Systemic lupus erythematosus (SLE) is a common autoimmune disease characterized by the development of autoantibodies to nucleic acid-containing cells. Systemic findings include lupus nephritis, dermatological, musculoskeletal or hematological manifestations [1]. Commonly described lesions in the eye include orbital vasculitis, myositis, keratoconjunctivitis sicca or retinal microangiopathy [2]. Lupus choroidopathy is a rare ocular manifestation, with approximately 40 cases reported worldwide [3], and generally occurs in association with lupus nephritis. The majority present with multifocal exudative retinal detachments which have classically been studied with clinical examination and fundus fluorescein angiography [4,5]. Optical coherence tomography (OCT) is a newer modality which is increasingly being employed in the management of these patients [6,7]. We report the systemic, clinical and OCT findings and an additional novel OCT finding of a bacillary detachment of a patient with severe lupus nephritis associated with SLE choroidopathy and the serial follow-up.

## Case presentation

A 55-year-old female presented to her local physician with a recent two-month history of bilateral lower limb swelling which had increased since the last three days, facial swelling and red facial rash along with a decreased frequency of micturition. Her previous significant medical history included systemic hypertension and hypothyroidism currently being treated. Baseline investigations revealed a fasting blood glucose of 73.8 mg/dl (normal: 60.0-150.00 mg/dl), an elevated serum creatinine of 2.56 mg/dl (normal: 0.6-1.20 mg/dl) and a blood urea nitrogen of 59.71 mg/dl (normal: 13.0-43.0 mg/dl). An abdominal ultrasound showed slightly altered renal cortical echotexture suggestive of medical renal disease. Mild fullness of the bilateral pelvicalyceal system and entire ureter was noted along with mild urothelial thickening noted along the entire pelvicalyceal system and ureters. Following a urological consult, she underwent a cystoscopy with urethrotomy with bilateral retrograde ureteropyelography (RUP) for suspected hydronephrosis.

A nephrologist’s opinion was sought, and subsequent investigations revealed a positive antinuclear antibodies (ANA) +++ test (immunofluorescence method; titer 1:10,000) and positive anti-double-stranded DNA (dsDNA) testing (98.62 IU/ml, normal: <30.0 IU/ml), and glomerular basement membrane (GBM) antibody testing was negative with equivocal test results for perinuclear antineutrophil cytoplasmic antibodies (P-ANCA) (myeloperoxidase (MPO)) (9.74 IU/ml, normal: <9.0 IU/ml) and negative results for cytoplasmic ANCA (c-ANCA) antibodies (0.88 IU/ml, normal: <3.50 IU/ml). A needle biopsy of the kidney revealed focal necrotizing, diffuse proliferative and crescentic glomerulonephritis with diffusely thickened capillary walls featuring predominantly cellular crescents over 3/36 of sampled glomeruli and tuft necrosis in 4/36 glomeruli. Direct immunofluorescence (DIF) studies revealed glomerular capillary and mesangial immune complex deposits (immune-mediated crescentic and diffuse proliferative glomerulonephritis). Patchy acute tubular injury is noted. A clinical diagnosis of type IV lupus nephritis with activity and chronicity was made.

At this time, an ophthalmic opinion was sought for her 15-day history of bilateral gross visual loss. Records reveal a visual acuity of 6/18 bilaterally with a diagnosis of bilateral multiple exudative retinal detachments. An optical coherence tomography (OCT) scan at the time reported retinal pigment epithelium (RPE) and serous retinal detachments bilaterally. Based on a confirmed diagnosis of systemic lupus erythematosus with lupus nephritis, she was treated with systemic steroids (prednisolone 60 mg/day) and intravenous cyclophosphamide (500 mg, single dose). Hemodialysis was initiated for persistent anuria and a raised serum creatinine of 6.4 mg/dl (normal: 0.6-1.20 mg/dl). She was administered three doses of methylprednisolone 500 mg intravenously, and she was transferred to our center.

On examination, she was pale with gross anasarca and bilateral pitting edema of the lower limbs. Vital parameters including her blood pressure (140/70 mmHg), heart rate (92/min) and oxygen saturation (98%) were normal. Examination of her respiratory and cardiovascular systems was normal. She was comprehensively investigated with routine hematological tests, renal and liver function tests. Relevant findings included mild anemia (8.2 g/dl; normal: 12.0-15.0 gm/dl) and leukocytosis (16,700/mm^3^; normal: 4,000-11,000/mm^3^) with a predominant neutrophilic response (absolute neutrophil count: 13,500/mm^3^; normal: 2,000-7,000/mm^3^). Her serum creatinine was 4.35 mg/dl (normal: 0.6-1.20 mg/dl). As she was mildly febrile, blood and urine cultures were sought, which grew *Escherichia coli*. The presence of a bloodstream infection precluded the use of further immunosuppression in the form of rituximab or calcineurin inhibitors.

**Figure 1:**
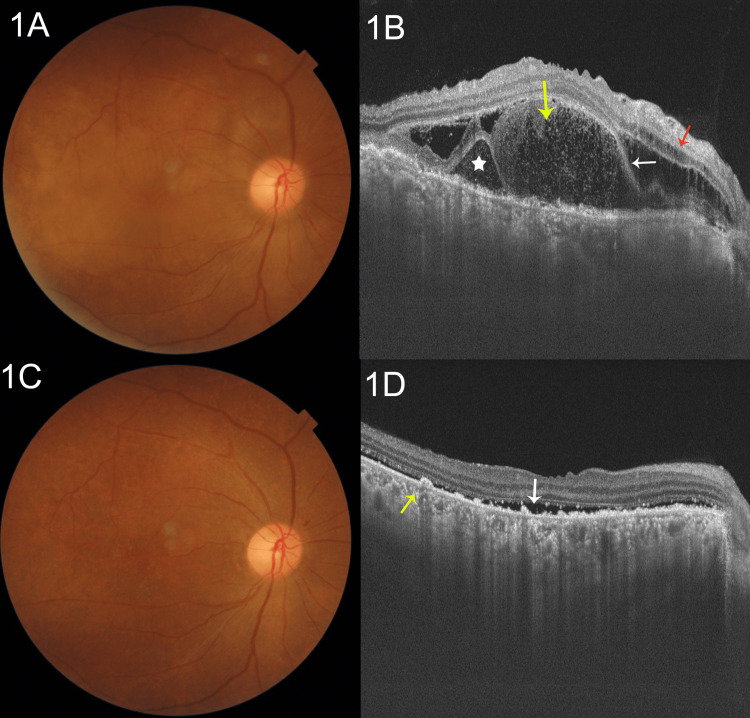
Clinical and OCT findings in the right eye (A) A dilated fundus examination revealed multiple yellowish lesions throughout the posterior pole consistent with pockets of subretinal fluid in the right eye. (B) A swept-source optical coherence tomography (OCT) reveals the presence of fluid in both the intraretinal and subretinal spaces. The white asterisk shows the presence of a retinal pigment epithelial (RPE) detachment. There is a presence of the bacillary layer detachment, with the accumulation of fluid in the intraretinal space indicated by the yellow arrow. The white arrow indicates the bacillary layer detachment, inferior to which is the subretinal fluid. The red arrow denotes the presence of cysts in the outer nuclear layer. (C) At her third follow-up, there was a near-complete regression of the exudative retinal detachments bilaterally with pigmentary changes. (D) The OCT scans revealed significant regression of the serous retinal and RPE detachments with a thin rim of residual subretinal fluid indicated by the white arrow. RPE hypertrophy, irregularity and bumps are indicated by the yellow arrow. The retinal layers were fully reattached.

**Figure 2:**
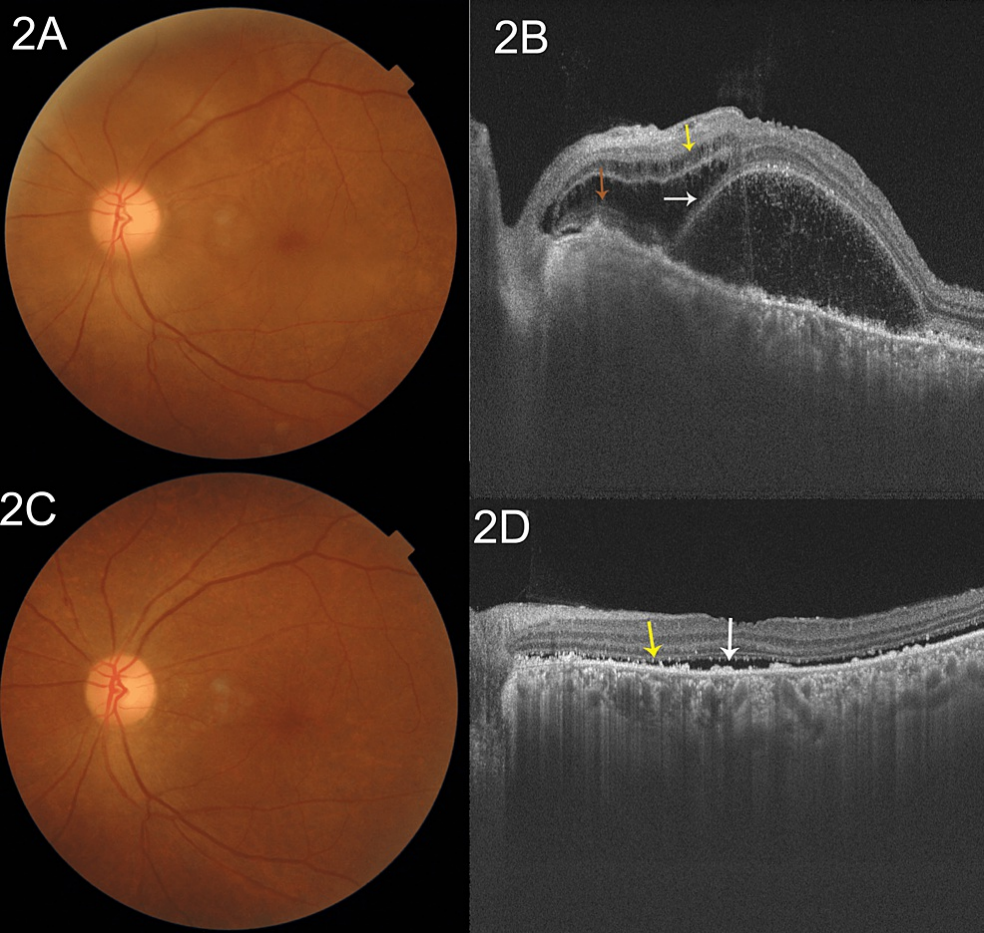
Clinical and OCT findings in the left eye (A) A dilated fundus examination revealed multiple yellowish lesions throughout the posterior pole consistent with pockets of subretinal fluid in the left eye. (B) A swept-source optical coherence tomography (OCT) reveals the presence of fluid in both the intraretinal and subretinal spaces. There is a presence of the bacillary layer detachment (white arrow), inferior to which is the subretinal fluid. The red arrow denotes the presence of outer retinal layers adherent to the retinal pigment epithelium (RPE)/Bruch's membrane layer. There is a presence of intraretinal fluid (yellow arrow). (C) At her third follow-up, there was a near-complete regression of the exudative retinal detachments bilaterally with pigmentary changes. (D) The OCT scans revealed significant regression of the serous retinal and RPE detachments with a thin rim of residual subretinal fluid indicated by the white arrow. RPE hypertrophy, irregularity and bumps are indicated by the yellow arrow. The retinal layers were fully reattached.

A detailed ophthalmic evaluation was performed. Her visual acuity was 6/36 improving to 6/18 in either eye. The appropriate presbyopic correction (+2.50 diopters) revealed her near vision to be N36 without any further improvement. A slit-lamp examination of either eye was normal as were her intraocular pressures and pupillary reactions. No evidence of anterior uveitis (cells/flare) or vitreous cells was detected. A dilated fundus examination revealed multiple yellowish lesions throughout the posterior pole consistent with pockets of subretinal fluid in the right eye (Figure 1A). A swept-source OCT was performed that revealed the presence of fluid in both the intraretinal and subretinal spaces. The white asterisk shows the presence of a retinal pigment epithelial (RPE) detachment. There is a presence of the bacillary layer detachment, with the accumulation of fluid in the intraretinal space indicated by the yellow arrow. The white arrow indicates the bacillary layer detachment, inferior to which is the subretinal fluid. The red arrow denotes the presence of cysts in the outer nuclear layer (Figure 1B). Similar fundus findings were seen in the left eye (Figure 2A). An OCT showed the presence of the bacillary layer detachment (white arrow) with the accumulation of fluid in the intraretinal space indicated by the yellow arrow. The red arrow denotes the presence of outer retinal layers adherent to the RPE/Bruch's membrane layer (Figure 2B). There is an apparent choroidal thickening bilaterally, but we were not able to quantify it further.

Vascular access was re-established via a right-sided internal jugular tunnel cuffed catheter (TCC), and a decision was made to employ plasmapheresis (PLEX) along with her routine thrice-weekly hemodialysis. Additionally, the systemic steroids were continued with the addition of hydroxychloroquine (200 mg/day) and cilnidipine (10 mg/day). She was monitored with visual acuity assessment, slit-lamp examination, dilated fundus examination and OCT scans weekly. At her third follow-up (day 22) following five sessions of plasmapheresis and continued immunosuppressive treatment, her vision had improved to 6/9 unaided bilaterally and to N6 with the appropriate correction. There was a near-complete regression of the exudative retinal detachments bilaterally with pigmentary changes (Figures 1C, 2C). The OCT scans revealed significant regression of the serous retinal and RPE detachments with a thin rim of residual subretinal fluid indicated by the white arrow. RPE hypertrophy, irregularity and bumps are indicated by the yellow arrow. The retinal layers were fully reattached (Figures 1D, 2D).

## Discussion

Systemic lupus erythematosus (SLE) is a multisystem autoimmune disease with protean manifestations. Several phenotypes exist, and patients may present with numerous clinical manifestations ranging from a mild dermatological involvement to severe multiorgan disease. The pathogenesis is complex and is currently thought to be due to the activation of autoimmunity in genetically susceptible patients. The underlying pathogenetic mechanism is an activation of T and B cells which then leads to cytokine release, complement activation and autoantibody production [8]. Lupus autoantibodies tend to attack nucleic acid-containing cells, and the subsequently formed antigen-antibody immune complexes may be deposited in the vascular beds of multiple organs including the eye [9].

Systemic manifestations include dermatological (malar rash), musculoskeletal (arthritis), hematological (cytopenia), cardiovascular (serositis, pneumonitis, interstitial lung disease), central nervous system (neuropsychiatric, optic neuritis, aseptic meningitis, demyelinating disease) and renal (lupus nephritis). SLE can affect virtually every tissue of the eye. Commonly described findings include as follows: A) Orbital manifestations: This includes a vasculitis that manifests as proptosis, painful orbital areas, defective vision and restriction of extraocular movements. Nonperfusion of the globe and extraocular muscles is common and may lead to optic nerve ischemia. Myositis may present similarly and is best diagnosed on computed tomography or ultrasound as an enlargement of one or several extraocular muscles. B) Ocular surface manifestations: The commonest ocular manifestation of SLE is keratoconjunctivitis sicca which occurs in the majority and is usually due to an aqueous layer deficiency. C) Scleral/corneal manifestations: These include a mild episcleritis or a phenotype of scleritis which typically presents with higher degrees of pain and may potentially be sight-threatening. Phenotypes of scleritis reported include anterior and posterior scleritis. D) Retinal manifestations: These may be seen in up to 29% of patients with active disease. Retinal microangiopathy consisting of small intraretinal hemorrhages and cotton wool spots is the commonest manifestation. Mechanisms of causation include immune complex deposition in the vessel wall with subsequent inflammation. A subset of patients present with retinal vasculitis with retinal venular or arteriolar inflammation. This is closely linked to the presence of antiphospholipid antibodies including anticardiolipin. Several reports have highlighted central retinal artery/vein occlusions or retinal ischemia as important causes of visual morbidity. E) Optic nerve lesions in the form of optic neuritis or ischemic optic neuropathy have been reported [2].

Lupus choroidopathy is a rare ocular manifestation with approximately 40 patients described till date in the literature [3]. In a recent study of among 161 patients with SLE, lupus choroidopathy was detected in only one patient (0.6%) [10]. It generally occurs in association with severe disease, especially lupus nephritis. Histopathological studies have revealed mononuclear inflammatory cells in the choroid. Fibrin deposition, a finding classically associated with vasculitis, may also be seen in the vessel wall and Bruch's membrane [9]. Uncontrolled hypertension may also have a role to play. Additionally, a thrombotic microangiopathy may occur that leads to choroidal hypoperfusion and subretinal leakage.

Nguyen and co-workers described the management of three cases of SLE choroidopathy and included a literature review which included 25 previously published papers [4]. These included 47 eyes of 28 patients. The cohort was largely female and had bilateral involvement, and systemic vascular disease, usually nephritis, was noted in all patients. Common phenotypes included an exudative retinal detachment which was seen in 10 (36%) patients followed by the serous detachment of the sensory retina seen in nine (32%). Less common findings included retinal pigment epithelium detachment seen in nine (32%), and the remainder of patients had retinal pigment epitheliopathy, serous detachment in the macular area, retinal hemorrhage or vasculitis. Angiographic correlates included fluorescein leakage commonly followed by a delayed choroidal filling or choriocapillaris ischemia. They suggest that SLE choroidopathy may have a greater prevalence than described and may strongly correlate to disease severity [4].

Jabs et al. described their experiences with six patients who presented with serous detachments of the retinal pigment epithelium and retina in a multifocal pattern that had its fluorescein angiographic correlates in several areas of late hyperfluorescence within the sensory retinal detachments [5]. Good systemic management resulted in the resolution of the ocular disease in three patients. The authors suggest that choroidal vascular lesions are the most likely pathogenetic mechanism [5]. 

These reviews were described prior to the widespread adoption of OCT in retinal practices. Kouprianoff et al. described the systemic, clinical and OCT findings of a 16-year-old female with SLE [6]. They noted bilateral neurosensory detachments in the macular area via OCT and monitored its progress over 17 months till its resolution [6]. Ozturk et al. described the findings in a 36-year-old female with bilateral findings including disc edema, retinal edema and serous retinal detachments [7]. OCT revealed intraretinal fluid-filled cavities and serous retinal detachment [7].

The patient we describe is a 55-year-old woman with a previous diagnosis of SLE, who developed lupus nephritis and bilateral severe gross visual loss. She was treated with immunosuppressives, hemodialysis and plasmapheresis (PLEX). PLEX probably works in several ways: it may remove autoantibodies and circulating immune complexes or may replace abnormal proteins. PLEX has so far been used to treat patients with severe neurological manifestations of SLE including psychosis or acute myelopathy. Available literature for the use of PLEX in the ocular manifestations of SLE is limited. Papadaki and co-workers have described the successful treatment of two patients with retinal vasculitis who were treated with PLEX and immunosuppression [11].

Clinical findings included RPE detachments, sub- and intraretinal fluid and bacillary detachments. A widespread choroidal vasculitis with consequent leakage into the subretinal and intraretinal space appears to account for our observed findings. Bacillary detachments are a novel OCT sign recently described that may denote a split between the myoid and ellipsoid layers of the inner photoreceptor segment usually following outer retinal lesions or infections/inflammations of the choroid. It has been reported in numerous conditions including toxoplasmic retinochoroiditis, Vogt-Koyanagi-Harada disease and chronic serous chorioretinopathy [12]. In our patient, similar findings of bacillary detachment are seen and follow the general pattern of a post-choroidal inflammatory finding. A systematic search on PubMed (www.pubmed.gov) using the search terms “bacillary layer detachment” and “systemic lupus erythematosus” returned no results and suggests that our findings constitute a novel finding and that the spectrum of bacillary layer detachment is expanded to include SLE choroidopathy.

## Conclusions

The patient we describe had classic clinical and investigational findings of SLE. The development of lupus nephritis with the consequent choroidal lesions led to a profound visual loss. Fundus examination and OCT studies established the diagnosis of SLE choroidopathy and guided its further management with systemic immunosuppression, hemodialysis and plasmapheresis. There was a rapid resolution of the retinal and choroidal findings with visual recovery over the next month.

SLE choroidopathy is an infrequently reported complication of SLE. Limited cases have been reported in the literature with the vast majority presenting with multifocal exudative retinal detachments with findings of intraretinal and subretinal fluid collections. Appropriate treatment usually results in good anatomical and visual recovery. The relatively new modality of OCT permitted us a good characterization of the retinal/choroidal disease and allowed us to describe a novel finding of bacillary layer detachment in this patient. Serial follow-up allowed us to monitor the resolution of the disease.
